# Thermosensitive Hydrogel Integrated with Bimetallic Nano‐Enzymes for Modulating the Microenvironment in Diabetic Wound Beds

**DOI:** 10.1002/advs.202411575

**Published:** 2024-12-16

**Authors:** Chuwei Zhang, Xiaoyi Zhang, Fei Li, Bo Li, Mengnan Zhang, Wanqian Li, Pan Zhuge, Jingye Yao, Yi Zhang, Shixuan Chen, Yongjin Fang, Chao Cai

**Affiliations:** ^1^ Department of Burn and Plastic Surgery Department of Wound Repair Surgery Affiliated Hospital of Nantong University Nantong Jiangsu 226001 China; ^2^ Zhejiang Engineering Research Center for Tissue Repair Materials Wenzhou Institute University of the Chinese Academy of Sciences Wenzhou Zhejiang 325000 China; ^3^ Office of Good Clinical Practice Affiliated Hospital of Nantong University Nantong, Jiangsu 226001 China; ^4^ Department of Otolaryngology Affiliated Jinhua Hospital Zhejiang University School of Medicine Jinhua Zhejiang 321000 China

**Keywords:** anti‐inflammation, diabetic wound healing, nano‐enzymes, thermo‐sensitive hydrogel

## Abstract

Effective regulation and reconstruction of the microenvironment are critical for the regeneration of chronic wounds. Diabetic wounds, in particular, pose a significant clinical challenge due to increased oxidative stress and dysfunctional healing processes. In this study, a novel therapeutic strategy is developed using 3D copper‐magnesium bimetallic antioxidant nano‐enzymes (Cu/Mg‐MOF) to mitigate reactive oxygen species (ROS) and restore redox balance through electron transfer. To optimize delivery, a thermo‐sensitive hydrogel composed of chitosan (CS) and *ε*‐polylysine (PL) is designed, serving as an efficient carrier for the nano‐enzymes. This Cu/Mg‐MOF@CS/PL hydrogel exhibits excellent physical properties, including injectability, softness, and biocompatibility, making it ideal for application in diabetic wounds. In a diabetic wound model, treatment with Cu/Mg‐MOF@CS/PL hydrogel significantly accelerated wound healing, with a closure rate of 90.6% by day 14, compared to just 55.4% in the untreated group. The hydrogel effectively promoted key aspects of wound healing, such as collagen deposition, re‐epithelialization, angiogenesis, and immunomodulation. These findings underscore the potential of the Cu/Mg‐MOF@CS/PL hydrogel as a promising therapeutic system for enhancing the healing of diabetic wounds.

## Graphical abstract

In this study, a novel copper‐magnesium bimetallic antioxidant nano‐enzymes (Cu/Mg‐MOF) loaded thermos hydrogel is developed to modulate the microenvironment in diabetic wound beds. It is capable of switching local inflammatory responses from a pro‐inflammatory status to a pro‐regenerative status. In addition, it can promote angiogenesis, collagen deposition, and epithelialization.

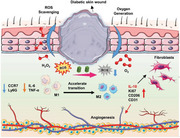

## 1 Introduction

Diabetic wound healing is severely limited by hyperglycemia, excessive exudate and reactive oxygen species (ROS), repeated infections, microcirculation disorders, and other problems, resulting in severe disability and mortality.^[^ ^1^ ^]^ Chronic high blood glucose levels lead to an increase in ROS and subsequent disruption of the redox balance at the wound site,^[^ ^2^ ^]^ which promotes inflammation and impairs cellular functions that are critical to the wound repair process,^[^ ^3^ ^]^ including cell migration and angiogenesis, ultimately impeding healing.^[^ ^4^ ^]^ The diminished synthesis of antioxidant enzymes at the site of injury in diabetic wounds is a significant factor causing this complication.^[^ ^5^ ^]^ Although natural antioxidant enzymes have a significant therapeutic effect in diabetic wound healing,^[^ ^6^ ^]^ their limited bioavailability, potential antigenicity, and low pathologic environmental stability restrict their widespread clinical application.^[^ ^7^ ^]^ In recent years, synthetic nanozymes have attracted much attention because of their advantages such as simple synthesis, substantial stability, and strong scalability.^[^ ^8^ ^]^ Studies have shown that copper (Cu) oxide–based nanozymes have been extensively researched in diabetic wound repair because these nanoparticles exhibit excellent ROS scavenging ability through mixed metal ion oxidation states.^[^ ^9^ ^]^ Moreover, Mg^2+^ and Cu^2+^, as essential elements of basic biochemical reactions in the human body,^[^ ^10^ ^]^ can promote cell proliferation and tissue regeneration,^[^ ^11^ ^]^ and play an important role in vascular regeneration and collagen deposition.^[^ ^12^ ^]^ Accordingly, Cu and Mg based nanozymes are expected to become potential competitors in the treatment of chronic nonhealing wounds.^[^ ^13^ ^]^ However, the preparation conditions of nanozymes are complex and variable, which is challenging for their production. Therefore, a low‐toxic, safe, and effective method to prepare nanozymes is urgently needed.

In order to avoid the problem of metal ion aggregation toxicity, a thermosensitive hydrogel (CS/PL gel) composed of chitosan (CS) and *ε*‐polylysine (PL) was developed as an ideal carrier.^[^ ^14^ ^]^ Chitosan is a kind of extensive source natural polysaccharide with beneficial properties such as low immunogenicity,^[^ ^15^ ^]^ good degradability,^[^ ^16^ ^]^ absorbability,^[^ ^17^ ^]^ and biocompatibility,^[^ ^18^ ^]^ which has been widely used in wound repair.^[^ ^19^ ^]^ *ε*‐Polylysine, as a natural biological metabolic product, exhibits good antibacterial ability and thermal stability,^[^ ^20^ ^]^ and has obvious effects as a food additive and wound repair material.^[^ ^21^ ^]^ Therefore, CS and PL were selected as the matrix materials of hydrogel, and the cross‐linking network was formed rapidly induced by body temperature.^[^ ^22^ ^]^

**Figure 1:**
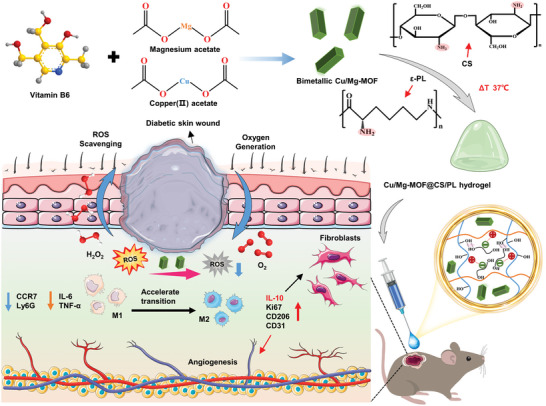
Preparation of a temperature‐sensitive injectable hydrogel incorporating Cu/Mg bimetallic MOFs, and the schematic illustration of its mechanism of modulating local angiogenesis and the inflammatory microenvironment in diabetic wounds.

In this research, the Cu/Mg‐MOF@CS/PL hydrogel was developed for microcirculation reconstruction in diabetic wounds (**Figure** **1**). We first prepared Cu/Mg‐MOF by solvothermal method, and the morphology and structural stability of the nanozymes were optimized by changing the concentration of Cu and Mg. Then, the optimized nanozyme was mixed with CS and PL precursor solution, and *β*‐Glycerol phosphate disodium salt (*β*‐GP) was used as the initiator. Notably, with the increase in temperature, energy is provided for electrostatic bonding and the formation of hydrogen bonds between molecules, and finally a composite hydrogel cross‐linking network is formed.^[^ ^23^ ^]^ The composite hydrogel (Cu/Mg‐MOF@CS/PL) exhibits good temperature sensitivity, softness, and injectability. When injected into the wound area, it quickly changes from liquid to hydrogel under the stimulation of body temperature. Further, to investigate the physical properties and in vitro therapeutic effects of Cu/Mg‐MOF@CS/PL hydrogel, and to verify its role in the repair of diabetic wounds, including accelerating re‐epithelialization, vascularization, collagen deposition, and regulation of inflammation. Herein, the preparation of a ROS responsive thermosensitive hydrogel is systematically reported and the effects on the microenvironment reconstruction of chronic wounds are presented.

## 2 Results and Discussion

### 2.1 Synthesis and Characterization of Cu/Mg‐MOF

**Figure 2:**
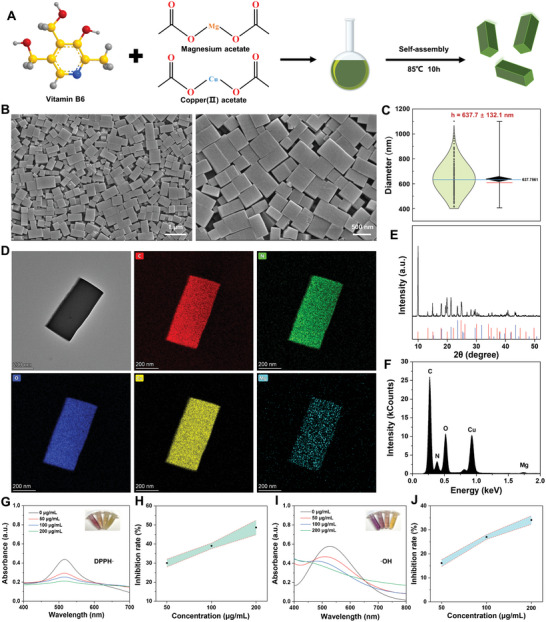
Synthesis and characterization of bimetallic Cu/Mg‐MOF prepared in ethylene glycol. A) Schematic diagram of the self‐assembly of bimetallic Cu/Mg‐MOF. B) SEM images of the bimetallic Cu/Mg‐MOF. C) Height distribution map of bimetallic Cu/Mg‐MOF. D) TEM and elemental mapping images of bimetallic Cu/Mg‐MOF. E) The X‐ray diffraction (XRD) patterns of bimetallic Cu/Mg‐MOF. F) Energy dispersive X‐Ray spectroscopy (EDS) analysis of the bimetallic Cu/Mg‐MOF. G, H) DPPH radicals scavenging ability of Cu/Mg‐MOF, the inner lining picture shows the color change of the solution after reaction with different concentrations of Cu/Mg‐MOF (0, 50, 100, and 200 µg mL^−1^). I, J) Hydroxyl radicals scavenging ability of Cu/Mg‐MOF, the inner lining picture shows the color change of the solution after reaction with different concentrations of Cu/Mg‐MOF (0, 50, 100, and 200 µg mL^−1^).

Recent studies have shown that nanocomposites based on Cu or Mg ions exhibit broad applications in tissue engineering.^[^ ^24^ ^]^ In this research, a self‐assembled nanozyme of Cu/Mg‐MOF was prepared in ethylene glycol using the solvothermal method, with copper and magnesium ions serving as connection points and Vitamin B6 as the framework (**Figure** **2**A). It is worth noting that we chose low‐toxic ethylene glycol as the solvent and prepared MOF under relatively mild conditions (85 °C). First, the morphology of MOF was regulated by changing the ratio of copper acetate and magnesium acetate. We observed that as the magnesium ion concentration increased, the color of the MOF progressively darkened (Figure S1, Supporting Information). Additionally, as illustrated in Figure S2 (Supporting Information), altering the magnesium ion concentration significantly impacted the morphology of the MOF. In the absence of magnesium ions, the MOF exhibited a flake‐like structure with irregular sizes. The resulting MOF structures were unstable at magnesium concentrations of 0.1 and 0.5 mmol. However, when the magnesium ion concentration was optimized at 0.05 mmol, SEM images revealed a well‐defined 3D cuboid structure with uniform size (Figure 2B). The height of the Cu/Mg‐MOF was ≈637.7 ± 132.1 nm (Figure 2C). As shown by TEM and elemental mapping images (Figure 2D), Cu and Mg ions are uniformly distributed in the synthesized Cu/Mg‐MOF cuboid. X‐ray diffraction (XRD) patterns revealed distinct diffraction peaks at 9.97°, 13.28°, 14.94°, 17.99°, 19.88°, 21.27°, 23.47°, and 24.83°, with minimal deviation, which corresponds to standard diffraction patterns (JCPDS card no. 21–0277 and 20–0683) (Figure 2E).^[^ ^25^ ^]^ The elemental composition of the Cu/Mg‐MOF was characterized by Energy Dispersive X‐ray Spectroscopy (EDS), indicating that the atomic ratios of C, N, O, Cu, and Mg were 55.34%, 6.47%, 18.04%, 19.37%, and 0.78%, respectively (Figure S3, Supporting Information). In the UV–vis absorption spectrum, Cu/Mg‐MOF showed prominent absorption peaks at 332 and 424 nm (Figure S4, Supporting Information). In summary, by adjusting the ratio of reactants, we successfully synthesized MOFs with varying morphologies and screened 3D nano‐enzymes with uniform structure, morphology, and size for further experiments. Furthermore, we evaluated the antioxidant activity of Cu/Mg‐MOF and conducted experiments on scavenging DPPH and OH free radicals. The experimental results showed that Cu/Mg‐MOF showed enhanced **·**OH and DPPH**·** scavenging capabilities with increasing nanozyme concentrations. When the concentrations of Cu/Mg‐MOF were 50, 100, and 200 µg mL^−1^, the scavenging efficiencies for DPPH free radicals were 30.04 ± 2.04%, 39.04 ± 1.48%, 48.69 ± 3.83%, and for OH free radicals were 16.14 ± 1.44%, 27 ± 0.98%, and 34.11 ± 1.77%, respectively. This obvious ROS scavenging effect of Cu/Mg‐MOF can be attributed to the strong electron transfer between hydroxyl ligands and copper ions, thereby achieving a dynamic redox balance.^[^ ^24^, ^26^ ^]^

### 2.2 Characterization of Temperature‐Sensitive Hydrogels

**Figure 3:**
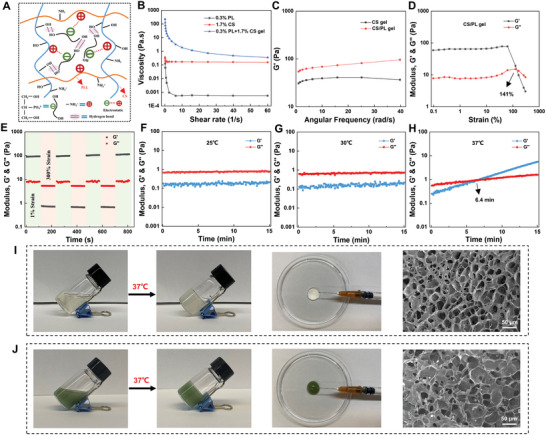
Characterization of temperature‐sensitive and injectable properties of the hydrogel. A) Schematic illustration of the mechanism of forming the hydrogel cross‐linked network. B) The viscosity of the formed hydrogel increase when PL was added to CS solution. C) Frequency sweep measurements of the CS and CS/PL hydrogel. The G′ of CS/PL hydrogel increase when PL was added to CS solution. D) Amplitude sweep measurements of the CS/PL hydrogel. The hydrogel network was disrupted when the strain was 141%. E) Continuous step strain sweep measurements of the CS/PL hydrogels. A small strain of 1% was followed by a large strain of 300% for 3.5 times. F–H) Constant‐temperature sweep of CS/PL hydrogel at 25, 30 and 37 °C. Temperature‐sensitive gel‐forming properties, injectability, and SEM images of the CS/PL hydrogel I) and CS/PL‐MOF hydrogel J). CS: chitosan; PL: *ε*‐polylysine.

**Figure** **3**A illustrates the formation mechanism of the thermosensitive hydrogels. Initially, water molecules and *β*‐glycerophosphate (*β*‐GP) form hydrogen bonds with the hydroxyl groups in chitosan (CS). Additionally, the ‐PO₄^3^⁻ groups in *β*‐GP electrostatically interact with the partially protonated amino groups (‐NH₃⁺) in CS and polylactide (PL), establishing molecular anchor points. The increase in temperature provides the necessary energy for these anchor points, facilitating the formation of a stable hydrogel cross‐linking network.^[^ ^27^ ^]^ Viscosity test results (Figure 3B) reveal that the viscosity of the CS/PL hydrogel is significantly higher than that of the CS or PL solutions alone. Figure 3C presents the storage modulus (Gʹ) of CS and CS/PL hydrogels across different angular frequencies, showing that the addition of PL increases the mechanical strength of the CS/PL hydrogel (95 Pa) compared to the CS hydrogel (38 Pa), attributed to the denser cross‐linking network. Strain‐amplitude scanning tests on the CS/PL hydrogel demonstrated that when the strain exceeded 141%, the hydrogel exhibited shear thinning behavior, causing the cross‐linking network to break down (Figure 3D). The self‐healing behavior of the CS/PL hydrogel was evaluated using a continuous strain method. At a low strain of 1% for 100 s, the hydrogel maintained its structural integrity and mechanical stability. However, the network collapsed at a high strain of 300% for 100 s, losing its mechanical stability. When the strain was reduced to 1%, the damaged hydrogel network rapidly recovered and reassembled (Figure 3E). After 3.5 testing cycles, both Gʹ (storage modulus) and Gʹʹ (loss modulus) returned to their initial values, indicating that the CS/PL hydrogel exhibits excellent self‐healing performance.^[^ ^28^ ^]^ To further confirm the thermal sensitivity, modulus measurements were conducted at 25, 30, and 37 °C, with sol‐gel transition times recorded (Figure 3F–H). The results demonstrated that the CS/PL hydrogel quickly transitioned into a gel at body temperature (37 °C), with an average sol‐gel transition time of 6.4 min. Additionally, both the CS/PL and Cu/Mg‐MOF@CS/PL hydrogels displayed favorable thermosensitive gelation and injectability. SEM images (Figure 3I,J) revealed that the hydrogels exhibited a 3D porous, loose structure, which supports their ability to fill irregular wound sites. These findings confirm that the CS/PL hydrogel possesses excellent injectability, softness, and temperature‐sensitive properties, making it an ideal candidate for applications in wound healing, especially for filling irregular wound geometries.

### 2.3 Protection against Exogenous Peroxidation Damage and Immunomodulatory Effect of Cu/Mg‐MOF and Cu/Mg‐MOF@CS/PL

**Figure 4:**
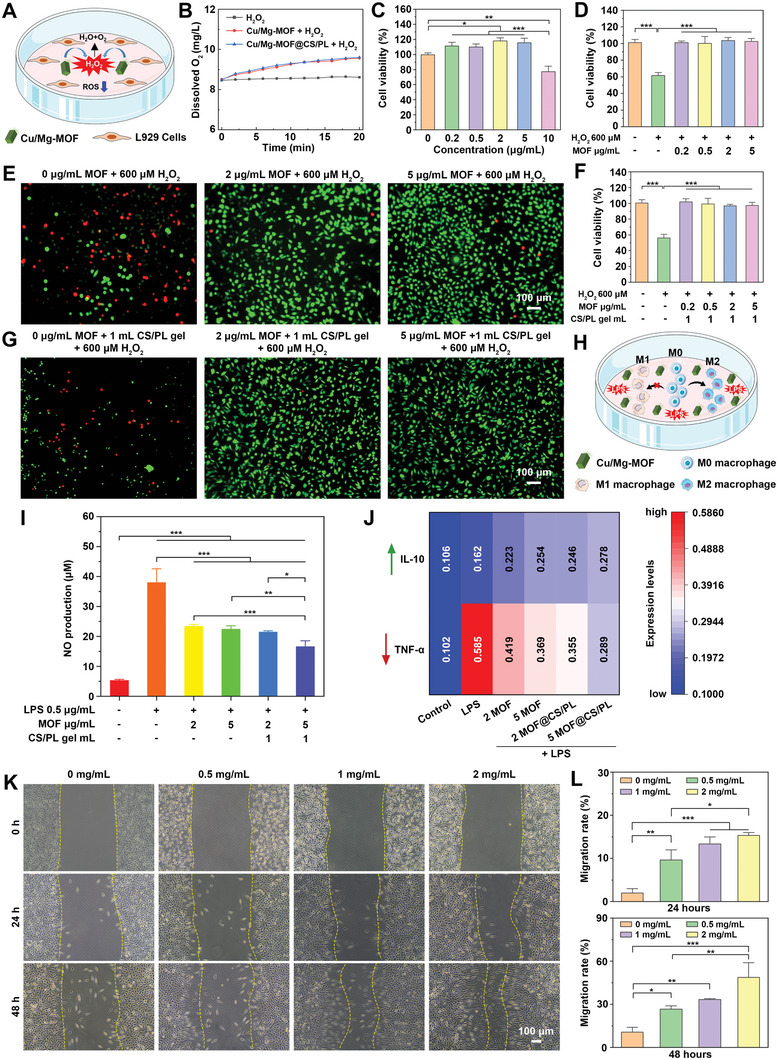
Cytocompatibility characterization and immunomodulatory effect of Cu/Mg‐MOF and Cu/Mg‐MOF@CS/PL hydrogel. A) Diagram of co‐incubation of L929 cells with Cu/Mg‐MOF stimulated by H_2_O_2_. B) H_2_O_2_ scavenging activity in vitro. C) Cell viability after incubation with different concentrations of Cu/Mg‐MOF for 24 h. D) Cell viability of untreated and Cu/Mg‐MOF‐treated L929 cells incubated with 600 µM H_2_O_2_. E) Representative live/dead staining of different concentrations of Cu/Mg‐MOF incubated with L929 cells under the stimulation of 600 µM H_2_O_2_. F) Cell viability of untreated and Cu/Mg‐MOF@CS/PL‐treated L929 cells incubated with 600 µM H_2_O_2_. G) Representative live/dead staining of different concentrations of Cu/Mg‐MOF@CS/PL incubated with L929 cells under the stimulation of 600 µM H_2_O_2_. H) Schematic illustration of co‐incubation of RAW 264.7 with Cu/Mg‐MOF stimulated by lipopolysaccharide (LPS). I) Cu/Mg‐MOF and Cu/Mg‐MOF@CS/PL induced NO production in LPS‐stimulated RAW 264.7 macrophages. J) The relative expression of pro‐inflammatory factors and anti‐inflammatory factors in RAW 264.7 after different treatments. K) Representative microscopic images of scratch wounds after L929 cells were co‐incubated with different concentrations of Cu/Mg‐MOF@CS/PL hydrogel extracts for 24 and 48 h, and quantification of cell migration rates L). ^*^ *p* < 0.05, ^**^ *p* < 0.01, ^***^ *p* < 0.001.

The toxicity of metal nanomaterials is considered a major challenge in their clinical applications.^[^ ^29^ ^]^ Therefore, the cytotoxicity of nanomaterials and composite hydrogels needs to be verified. First, we studied the sustained release characteristics of copper and magnesium ions in the Cu/Mg‐MOF@CS/PL hydrogel system. The experimental results showed that copper and magnesium ions were slowly released in the hydrogel system without explosive release. The dynamic equilibrium of release was basically reached on the 7th day, and the release rates of copper and magnesium ions were 65.53 ± 3.55% and 7.17 ± 0.23%, respectively (Figure S5, Supporting Information). **Figure** **4**A describes the regulatory effect of Cu/Mg‐MOF on mouse skin fibroblasts (L929) under the action of H_2_O_2_.^[^ ^12^ ^]^ We first investigated the scavenging effect of the Cu/Mg‐MOF and Cu/Mg‐MOF@CS/PL on hydrogen peroxide. The experimental results indicated that Cu/Mg‐MOF and Cu/Mg‐MOF@CS/PL have similar H_2_O_2_ decomposition efficiency and exhibited catalase‐like activity (Figure 4B). Next, the concentration‐dependent cytotoxicity of Cu/Mg‐MOF was tested. Figure 4C shows that after co‐culture at different concentrations (0.2, 0.5, 2, and 5 µg mL^−1^) for 24 h, Cu/Mg‐MOF promoted the proliferation of mouse skin fibroblasts (L929), while when the concentration was 10 µg mL^−1^, the cell activity was significantly reduced to 82.7 ± 9.2%. Fibroblasts are the main functional cells involved in wound healing; however, excessive ROS in the diabetic wound environment can impair cell activity. L929 cells exhibited oxidative stress death when exposed to high levels of exogenous H_2_O_2_ (600 µM), with cell viability reduced to 66.6 ± 7.4%, while the addition Cu/Mg‐MOF and Cu/Mg‐MOF@CS/PL was able to completely protect cells from 600 µM H_2_O_2_‐induced peroxidative death, and the cell activity was maintained above 90% (Figure 4D,F). Live/dead staining experiments further supported the peroxidative protection of Cu/Mg‐MOF and Cu/Mg‐MOF@CS/PL (Figure 4E,G). There were more dead cells exposed to 600 µM H_2_O_2_, while the number of dead cells was significantly reduced after protection by Cu/Mg‐MOF and Cu/Mg‐MOF@CS/PL. To investigate the inmmunomodulatory ability of Cu/Mg‐MOF and Cu/Mg‐MOF@CS/PL on macrophages, we used RAW 264.7 as a cell model and stimulated it with lipopolysaccharide (LPS) to induce a pro‐inflammatory M1 phenotype (Figure 4H). The experimental results showed that this simulation was resisted and the expression of ROS was significantly reduced when pretreated with Cu/Mg‐MOF and Cu/Mg‐MOF@CS/PL (Figure S6, Supporting Information). As shown in Figure 4I, co‐incubation of RAW 264.7 with Cu/Mg‐MOF and Cu/Mg‐MOF@CS/PL significantly reduced the level of NO production, which is a key indicator of cellular inflammatory status. In addition, pretreatment of RAW 264.7 with Cu/Mg‐MOF and Cu/Mg‐MOF@CS/PL can effectively alleviate cellular inflammatory response, reduce the expression of pro‐inflammatory factors, and increase the level of anti‐inflammatory factors (Figure 4J). Finally, we soaked the freeze‐dried Cu/Mg‐MOF@CS/PL hydrogel for 24 h to prepare extracts of different concentrations.^[^ ^30^ ^]^ The CCK‐8 experiment (Figure S7, Supporting Information) and live‐dead staining experiment (Figure S8,Supporting Information) verified that the Cu/Mg‐MOF@CS/PL hydrogel had good cell compatibility. The cell scratch experiment further demonstrated that the Cu/Mg‐MOF@CS/PL hydrogel promoted the migration of L929 cells. With the increase in incubation time, the cells in the Cu/Mg‐MOF@CS/PL hydrogel extract group migrated significantly (Figure 4K,L). After incubation for 48 h, the cell migration rate of the 2 mg mL^−1^ hydrogel extract group reached 48.67 ± 10.02%. These experimental results prove that Cu/Mg‐MOF@CS/PL hydrogel exhibits the ability to protect cells from exogenous oxidative stress, regulate inflammatory responses, and promote cell migration, which is beneficial for wound repair.

### 2.4 Diabetic Skin Wound Healing Assessment In Vivo

**Figure 5:**
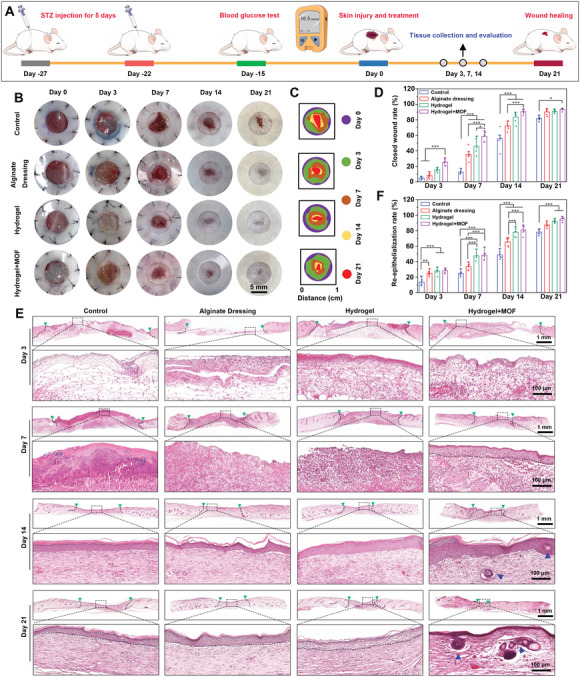
In vivo diabetic wound healing evaluation. A) Schematic illustration of mouse diabetic wound modeling. The blood glucose concentration should be maintained above 16.5 mmol L^−1^, and 3 weeks was allowed for the skin to develop glycosylation lesions. B) Representative photographs of diabetic skin wounds of Control, Alginate Dressing (commercially available medical wound dressing), Hydrogel, and Hydrogel + MOF groups at each indicated time. C) Schematic diagram showing the closed wound area from different time points. D) The wound closure rate of each group at 3, 7, 14, and 21 days. E) Representative H&E staining images of diabetic wound area at 3, 7, 14, and 21 days. The green arrows represent the edges of the wound and the blue arrows represent the new hair follicles. F) The re‐epithelialization rate of each group at 3, 7, 14, and 21 days. ^*^ *p* < 0.05, ^**^ *P* < 0.01, ^***^ *p* < 0.001.

A diabetic skin wound model in mice was established to assess the healing effects of Cu/Mg‐MOF@CS/PL hydrogels (**Figure** **5**A).^[^ ^31^ ^]^ Compared with the control group, the Alginate Dressing, CS/PL hydrogel, and Cu/Mg‐MOF@CS/PL hydrogel (Hydrogel + MOF) groups showed significantly accelerated wound healing at various time points (Days 3, 7, 14, and 21), with the Cu/Mg‐MOF@CS/PL hydrogel group demonstrating the most effective results (Figures 5B,C). During the early treatment period (Days 7 and 14), the Cu/Mg‐MOF@CS/PL hydrogel group had minimal residual wound areas. The wound closure rates for this group were 58.45 ± 5.9% on Day 7 and 90.6 ± 3.3% on Day 14, significantly surpassing the other groups (Figure 5D). Histological analysis using H&E staining (Figure 5E) further confirmed these results. By Day 7, partial epidermis formation was observed in the Cu/Mg‐MOF@CS/PL hydrogel group, while the control group still showed blood clots, indicating ongoing severe inflammation. By Day 14, new hair follicles appeared in the Cu/Mg‐MOF@CS/PL hydrogel group, suggesting superior recovery. By Day 21, the epidermal thickness in the Cu/Mg‐MOF@CS/PL hydrogel group had nearly returned to a healthy level, whereas the other groups still exhibited thicker‐than‐normal epidermis. As shown in Figure 5F, the re‐epithelialization rate in the Cu/Mg‐MOF@CS/PL hydrogel group reached 81.1 ± 6% by Day 14, and almost complete re‐epithelialization was observed on Day 21 (95.1 ± 2.9%). These results demonstrate that the Cu/Mg‐MOF@CS/PL hydrogel not only accelerated wound healing but also improved the overall quality of skin regeneration.

### 2.5 Evaluation of Proliferative Phase Activity in the Skin Defects

**Figure 6:**
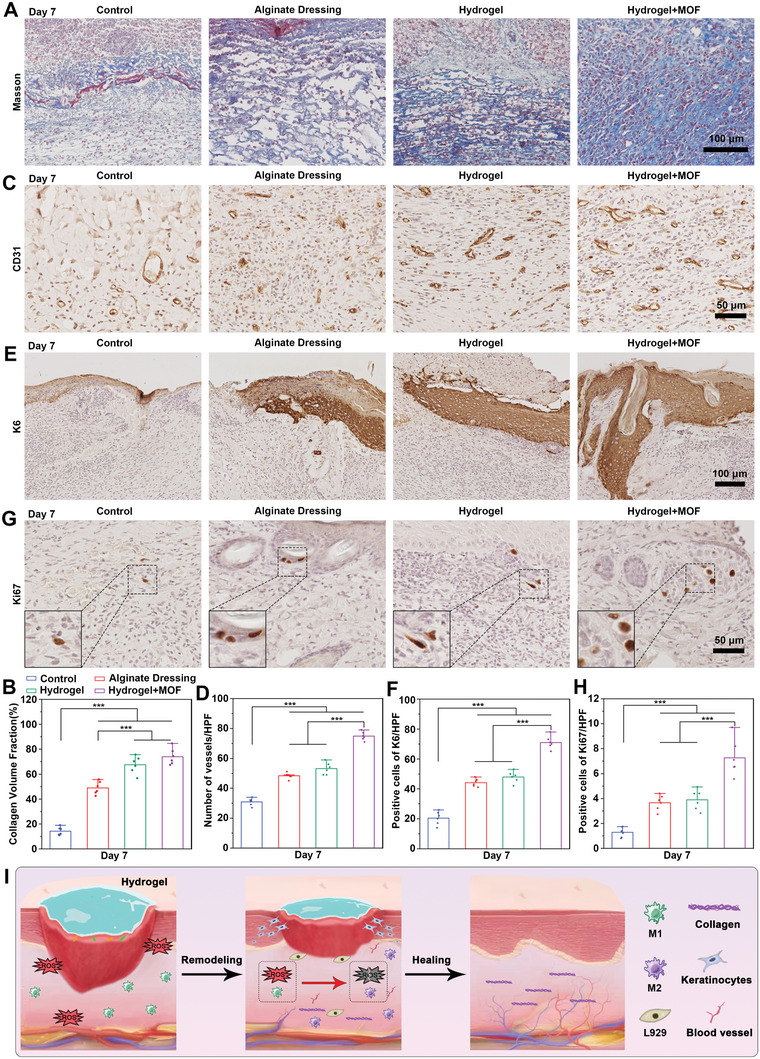
Evaluation of proliferative phase activity in the skin defects. A, B) Collagen deposition in the wound center area of control, alginate, hydrogel, and hydrogel + MOFs groups after 7 days of treatment. C, D) The expression of CD31 in the wound center area of control, alginate, hydrogel, and hydrogel + MOFs groups after 7 days of treatment. E, F) The expression of keratin 6 (K6) at the edge epidermis of control, alginate, hydrogel, and hydrogel + MOFs groups after 7 days of treatment. G, H) The expression of Ki67 in the wound center area of control, alginate, hydrogel, and hydrogel + MOFs groups after 7 days of treatment. I) Schematic illustration of Cu/Mg‐MOF@CS/PL hydrogel promoting angiogenesis, collagen deposition, and epithelialization. ^***^ *p* < 0.001.

Representative images of Masson staining are shown in **Figure** **6**A, where collagen fibers are stained blue, while myofibers and cytoplasm are stained red. Collagen is a major structural protein in the skin and plays a key role in the wound‐healing process. At the early stage of wound healing (Day 7), collagen deposition in the Cu/Mg‐MOF@CS/PL hydrogel group was significantly higher than in the other three groups, with a collagen volume fraction of 74.03 ± 6.6% (Figure 6B). In the later stage of wound healing, the blue collagen fibers were lacking in the control group, alginate dressing group, and hydrogel group, while the collagen deposition in the hydrogel + MOF group was denser, with a collagen deposition rate of 85.33 ± 3.2% (Figure S9, Supporting Information). In addition, CD31 staining revealed that the density of new blood vessels in the wound area was highest after 7 days of Cu/Mg‐MOF@CS/PL hydrogel treatment. This was accompanied by significantly increased infiltration of endothelial cells (CD31 positive cells). The proportion of CD31‐positive cells reached 74.8 ± 2.8%, significantly surpassing that of the other three groups (Figure 6C,D). Keratin 6 is a marker for the hyperproliferation of keratinocytes. The expression of K6 was markedly increased in the Cu/Mg‐MOF@CS/PL hydrogel group (71 ± 4.3%) compared to the other groups (Figure 6E,F). Additionally, the proliferation marker Ki67, essential for cell proliferation, showed consistently high expression levels in the Cu/Mg‐MOF@CS/PL hydrogel group, reflecting its role in promoting cellular growth (Figure 6G,H). These findings collectively demonstrate that the Cu/Mg‐MOF@CS/PL hydrogel provides significant advantages in accelerating wound healing by promoting keratinocyte and fibroblast proliferation, collagen deposition, and microvascular regeneration, thereby enhancing the reconstruction of the wound microenvironment. Based on the above results, this schematic shows that the Cu/Mg‐MOF@CS/PL hydrogel can accelerate the reconstruction of the wound microenvironment by promoting angiogenesis, collagen deposition, and epithelial regeneration (Figure 6I).

### 2.6 The Inflammation Regulation Effect of Cu/Mg‐MOF@CS/PL Hydrogel

**Figure 7:**
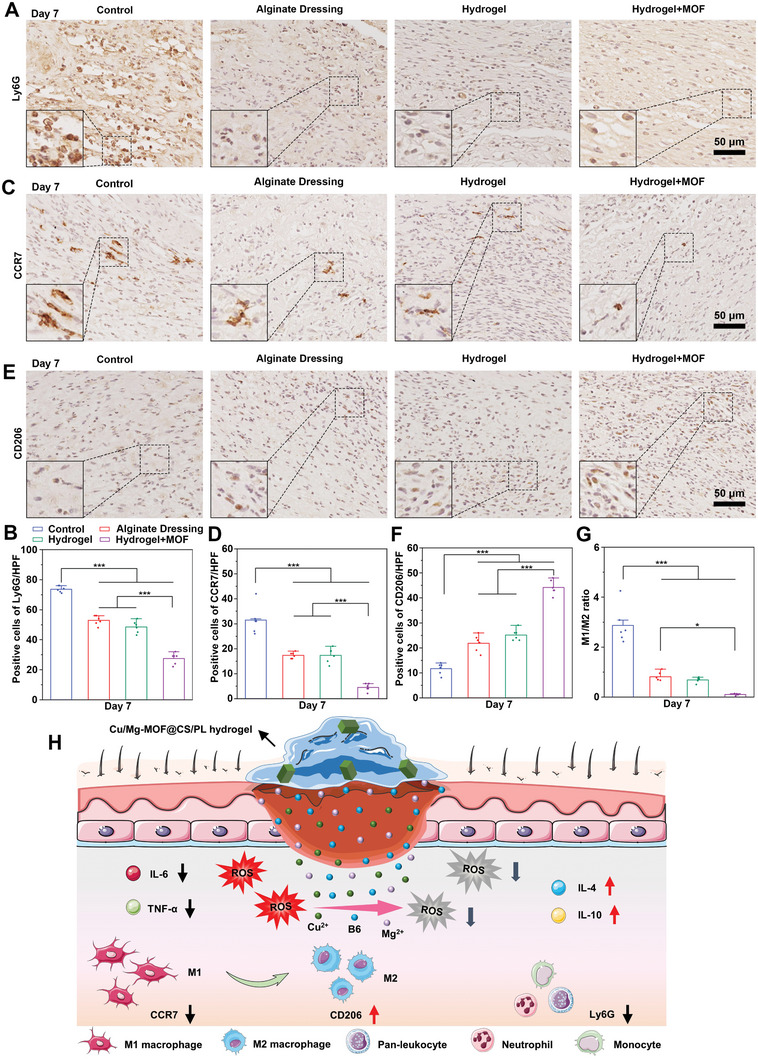
The assessment of local inflammatory responses. A, B) The expression of Ly6G (A pan marker for monocytes, granulocytes, and neutrophils) in the wound center area of control, alginate, hydrogel, and hydrogel + MOFs groups after 7 days of treatment. C, D) The expression of CCR7 (M1 type macrophage) in the wound center area of control, alginate, hydrogel, and hydrogel + MOFs groups after 7 days of treatment. E, F) The expression of CD206 (M2 type macrophage) in the wound center area of control, alginate, hydrogel, and hydrogel + MOFs groups after 7 days of treatment. G) The ratio between the numbers of M1‐type macrophages and the numbers of M2‐type macrophages in the control, alginate, hydrogel, and hydrogel + MOFs groups on day 7. H) The response of Cu/Mg‐MOF@CS/PL hydrogel to inflammatory cells and inflammatory cytokines. ^*^ *p* < 0.05, ^***^ *p* < 0.001.

**Figure 8:**
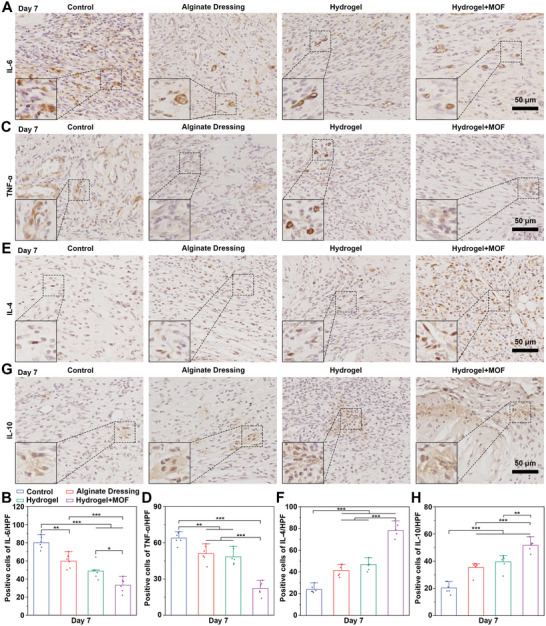
Characterization of inflammatory and anti‐inflammatory factors in early wound healing. A, B) The expression of IL‐6 in the wound center area of control, alginate, hydrogel, and hydrogel + MOFs groups after 7 days of treatment. C, D) The expression of TNF‐α in the wound center area of control, alginate, hydrogel, and hydrogel + MOFs groups after 7 days of treatment. E, F) The expression of IL‐4 in the wound center area of control, alginate, hydrogel, and hydrogel + MOFs groups after 7 days of treatment. G, H) The expression of IL‐10 in the wound center area of control, alginate, hydrogel, and hydrogel + MOFs groups after 7 days of treatment. ^**^ *P* < 0.01, ^***^ *P* < 0.001.

Considering the reactive oxygen species (ROS) scavenging ability of Cu/Mg‐MOF, we further explored the anti‐inflammatory properties of the Cu/Mg‐MOF@CS/PL hydrogel. As shown in **Figure** **7**A, wounds treated with the Cu/Mg‐MOF@CS/PL hydrogel exhibited significantly reduced expression of Ly6G, a marker for monocytes, granulocytes, and neutrophils, compared to those treated with the CS/PL hydrogel. Indicating a marked reduction in the presence of inflammatory cells. Quantitative data analysis revealed that the proportion of Ly6G‐positive cells in the Cu/Mg‐MOF@CS/PL hydrogel group was 27.5 ± 3.7%, notably lower than in the CS/PL hydrogel group (48.5 ± 4%) and the Alginate Dressing group (52.8 ± 3.1%) (Figure 7B). Moreover, the Cu/Mg‐MOF@CS/PL hydrogel also modulated macrophage polarization. The expression of CCR7, a marker for pro‐inflammatory M1 macrophages, was significantly reduced in wounds treated with Cu/Mg‐MOF@CS/PL hydrogel (Figure 7C,D), while the expression of CD206, a marker for anti‐inflammatory M2 macrophages, was significantly elevated (Figure 7E,F). By day 7, the ratio of M1 to M2 macrophages in the Cu/Mg‐MOF@CS/PL hydrogel group was less than 1, indicating a shift from a pro‐inflammatory state to a pro‐regenerative state (Figure 7G). In addition, we further detected the expression of inflammatory and anti‐inflammatory factors in the wound area on the 7th day. The results indicated that the expression of IL‐6 and TNF‐α in the wound treated with Cu/Mg‐MOF@CS/PL hydrogel was significantly lower than that in the other groups (**Figure** **8**A–D). On the contrary, the expression of IL‐4 and IL‐10 in the Cu/Mg‐MOF@CS/PL hydrogel group was significantly higher than that in the other groups (Figure 8E–H). This is mainly attributed to the fact that Cu/Mg‐MOF@CS/PL hydrogel down‐regulates the oxidative stress state in the wound area, relieves excessive inflammation, and reduces the expression of pro‐inflammatory factors (Figure 7H).

## 3 Conclusion

In this study, we developed a Cu/Mg‐MOF composite thermo‐sensitive hydrogel that significantly improved the microenvironment of chronic wound beds and then promoted diabetic wound healing. The nano‐enzymes, characterized by uniform morphology, structure, and size, were incorporated into a CS/PL‐based injectable hydrogel with excellent biocompatibility, allowing for rapid and effective wound coverage. Our results demonstrate that the Cu/Mg‐MOF@CS/PL hydrogel exhibits strong anti‐inflammatory properties, accelerates wound healing, and promotes the regeneration of skin appendages. This hydrogel system not only improves the overall quality of tissue repair but also supports the reconstruction of the microenvironment of chronic wound beds, providing a promising avenue for future research in tissue engineering and regenerative medicine.

## 4 Experimental Section

### Materials

Vitamin B6 (catalog number: 65‐23‐2), ethylene glycol (catalog number: 107‐21‐1), iron sulfate heptahydrate (catalog number: 7782‐63‐0), salicylic acid (catalog number: 69‐72‐7) and 1,1‐Diphenyl‐2‐picrylhydrazyl Free Radical (catalog number: 1898‐66‐4) were purchased from Aladdin (Shanghai, China). Magnesium acetate (catalog number: 142‐72‐3), copper acetate (catalog number: 6046‐93‐1), and hydrogen peroxide (30%, catalog number: 7722‐84‐1) were purchased from Sigma–Aldrich (St. Louis, USA). Chitosan (CS) (deacetylation degree 90%, 200–400 mPa s, catalog number: 9012‐76‐4) was purchased from Aladdin Biochemical Technology Co. Ltd. (Shanghai, China). 𝛽‐Glycerol phosphate disodium salt pentahydrate (𝛽‐GP·5H_2_O, catalog number: 13408‐09‐8) was purchased from Sigma–Aldrich (St. Louis, USA). *ε*‐polylysine (Mw = 2000−5000 Da, catalog number: 26700‐39‐0) was purchased from Aladdin (China). Dulbecco's Modified Eagles Medium (DMEM, catalog number: 11965118), Fetal bovine serum (FBS, catalog number: 10099–141C), and Penicillin‐Streptomycin (PS, catalog number: 15140122) were obtained from Gibco (Shanghai, China). Lipopolysaccharides (catalog number: 93572‐42‐0) were purchased from Sigma–Aldrich (St. Louis, USA). Antibody against CD31 (catalog number: ab28364) was purchased from Proteintech, antibody against CD206 (catalog number: PA5‐101657) was acquired from Invitrogen, antibody against CCR7 (catalog number: ab253187), K6 (catalog number: bsm‐60235R), and Ki67 (catalog number: ab16667) were acquired from Abcam (Shanghai, China), antibody against Ly6G (catalog number: 551459) was acquired from BD pharmingen.

### Synthesis of Copper/Magnesium‐Based Organic Framework (Cu/Mg‐MOF)

First, vitamin B6 was dissolved in ethylene glycol solution (20 mL), heated, and stirred at 85 °C for 10 min. Then, the Copper (II) acetate and Magnesium acetate were dissolved in an ethylene glycol solution (20 mL) at 85 °C. The dissolved copper (II) acetate and magnesium acetate solutions were quickly added to the vitamin B6 solution and stirred at a speed of 1000 rpm. After stirring for 10 h, centrifuge at 6000 rpm, and then wash with DI water and absolute ethanol three times respectively. Finally, the dark green powder was prepared by freeze‐drying method and stored at 4 °C. Among them, the ratio of vitamin B6:Cu:Mg was 1:0.5:0, 1:0.5:0.05, 1:0.5:0.1, 1:0.5:0.5 (mmol), respectively.

### Synthesis of Cu/Mg‐MOF@CS/PL Hydrogel

The chitosan (90% deacetylation degree) was dissolved in 0.1 M HCl solution, where the mass fraction of chitosan was 2.5%, disinfected at 121 °C for ≈15–20 min, and stored at 4 °C for later use. The PL solution (Wt% = 1.5%) was prepared with DI water and fully stirred at room temperature for ≈1 h to completely dissolve it. The bacteria were removed with a filter membrane (0.22 µm) and stored at 4 °C. Sodium *β‐*glycerophosphate solution (Wt% = 50%) was prepared with DI water, fully stirred at room temperature for ≈2 h to completely dissolve, sterilized with filter membrane (0.22 µm), and stored at 4 °C. Dissolve MOF into the mixed chitosan and PL solution, stir continuously for 10 min, and then slowly add *β‐*GP solution. The volume ratio of CS, PL, and *β‐*GP was 1:0.3:0.2. The whole process was best carried out under slow stirring, and the duration was ≈10–15 min. The above evenly mixed liquid was incubated at 37 °C to form a hydrogel.

### Measurement of Rheological Properties of Hydrogels

The rheological properties of hydrogels were measured using a rotating rheometer (DHR‐2, TA, USA). A metal parallel plate rotor with a diameter of 20 mm was used. The viscosity test parameters were set as follows: Gap Size = 1000 µm, Shear Rate = 0.1–60 s^−1^, Temperature = 25 °C. The mechanical properties of hydrogels were characterized by frequency scanning and strain scanning. The test parameters of the frequency sweep were set as follows: Gap Size = 1000 µm, Frequency = 1–40 rad s^−1^, Strain = 2%, Temperature = 25 °C. The test parameters of the strain sweep were set as follows: Gap Size = 1000 µm, Frequency = 2 rad s^−1^, Strain = 0.1–00%, Temperature = 25 °C. Step strain scanning measurement parameters were set as follows: Low strain (γ = 1.0%), High strain (γ = 350%), Frequency = 2 rad s^−1^, each strain interval of 100 s, repeated 3.5 times. The thermostatic scanning test parameters were set as follows: Gap Size = 1000 µm, Frequency = 2 rad s^−1^, Strain = 2%, and temperature 25, 30, and 37 °C, respectively.

### Release of Cu/Mg‐MOF from Cu/Mg‐MOF@CS/PL Hydrogel

ICP‐MS was used to quantitatively analyze the release of Cu/Mg‐MOF from the composite hydrogel. Briefly, the freeze‐dried hydrogel containing Cu/Mg‐MOF (0.6 mg) was immersed in 20 mL PBS (pH 7.4) and incubated in a 37 °C constant temperature shaker. At the specified time points (1, 2, 3, 4, 5, 6, 7 days), 50 µL of the solution was taken to detect the release of Cu/Mg‐MOF, and an equal volume of fresh PBS was added to maintain a constant volume.

### OH Elimination

The salicylic acid (SA) method was used to determine the scavenging ability of Cu/Mg‐MOF nanozymes for hydroxyl free radicals. After salicylic acid was dissolved in ethanol, it was introduced into the water together with Fe^2+^ and H_2_O_2_, and the final concentrations of SA, Fe^2+,^ and H_2_O_2_ were 0.6, 0.6, and 0.58 mM, respectively. The concentrations of Cu/Mg‐MOF nanozymes were 0, 50, 100, and 200 µg mL^−1^. The mixed system was incubated at 37 °C for 30 min, and the absorbance at 510 nm was measured.

### DPPH Elimination

Prepare 0.1 mM 1,1‐Diphenyl‐2‐picrylhydrazyl radical (DPPH) solution with anhydrous ethanol and store it in the dark. Place 1 mL of the sample solution and 1 mL of DPPH solution in the same test tube and mix well, then incubate at 37 °C for 30 min. Finally, measure the absorbance of the solution at 517 nm. The concentrations of nanozymes included were 0, 50, 100, and 200 µg mL^−1^ respectively.

### Scavenging effect of Cu/Mg‐MOF on H_2_O_2_

The Cu/Mg‐MOF was added to 1 mM H_2_O_2_ solution, and the final concentration of Cu/Mg‐MOF was 0.05 mg mL^−1^. In addition, composite hydrogels (Cu/Mg‐MOF@CS/PL) containing the same Cu/Mg‐MOF concentration were prepared. A dissolved oxygen meter (JPB‐80A, TUOHE, Guangzhou, China) was used to monitor oxygen solubility at 2 min intervals.

### Evaluation of Cell Viability and Intracellular ROS Elimination

L929 cells were seeded in 96‐well plates at 1 × 10^4^ cells/well. After overnight, different concentrations of Cu/Mg‐MOF (0.2, 0.5, 2, 5, and 10 µg mL^−1^) were added for co‐culture for 24 h. Cell proliferation rate and cell viability were detected using CCK‐8 assay (Beyotime, Shanghai, China). Meanwhile, L929 cells were seeded in 96‐well plates (1 × 10^4^ cells/well), and 48‐well plates (2 × 10^4^ cells/well). After overnight, different concentrations of Cu/Mg‐MOF (0.2, 0.5, 2, and 5 µg mL^−1^) and Cu/Mg‐MOF@CS/PL (containing 0.2, 0.5, 2, and 5 µg mL^−1^ of Cu/Mg‐MOF) were added and then stimulated with 600 µM H_2_O_2_. After 24 h of culture, cell viability was detected using CCK‐8, and live/dead fluorescence images were taken.

### Evaluation of Anti‐Inflammatory Effect

RAW 264.7 macrophages were seeded in 48‐well plates (5 × 10^4^ cells/well). First, cells were stimulated with 200 µM H_2_O_2_ for 24 h, then Cu/Mg‐MOF and Cu/Mg‐MOF@CS/PL were added to the cells for 24 h. The ROS level in the cells was detected using the ROS‐specific probe DCFH‐DA (1: 2000, Beyotime, Shanghai, China), and fluorescence images were taken. RAW 264.7 macrophages were seeded in 96‐well plates (10^4^ cells/well). The cells were stimulated with 0.5 µg mL^−1^ LPS for 24 h, then Cu/Mg‐MOF and Cu/Mg‐MOF@CS/PL were added to the cells for 24 h. The NO detection kit (Beyotime, Shanghai, China) was used to detect the NO content in the cell supernatant, and the absorbance was read at 550 nm. The ELISA kit (Beyotime, Shanghai, China) was used to detect the content of interleukin (IL)‐10 and tumor necrosis factor (TNF‐α) in the cell supernatant.

### Cell Viability and Cell Migration Detection of Cu/Mg‐MOF@CS/PL Hydrogel Extract

L929 cells were seeded on 96‐well plates at 1 × 10^4^ cells/well. After overnight, extracts of different concentrations were added and co‐cultured for 24 h. Among them, the content of Cu/Mg‐MOF in the freeze‐dried Cu/Mg‐MOF@CS/PL hydrogel was 5 µg mL^−1^. The initial extract was DMEM medium containing 1% FBS and the immersion time was 24 h. Cell proliferation rate and cell viability were detected by CCK‐8 analysis (Beyotime, Shanghai, China), and Live/Dead fluorescence images were taken. In addition, the migration behavior of L929 cells was further studied by scratch test. L929 cells were seeded in 6‐well plates at 6 × 10^5^ cells/well and cultured overnight to form a single confluent layer. Scratches were made with a 100 µL needle tip, washed 3 times with PBS, and photographed. L929 cells were then treated with Cu/Mg‐MOF@CS/PL hydrogel extracts of different concentrations for different time periods. At the specified time points, the closure of the scratches was observed and imaged.

### Diabetic Wound Model and Treatment

Male ICR mice (30–35 g) were given gavage before modeling (WIUCAS24022607). A type I diabetic mouse model was prepared by intraperitoneal injection of 40 mg kg^−1^ STZ for 5 days. After 1 week, the fasting blood glucose level was measured by a glucose meter (Roche Diagnostics, Shanghai, China), and the results were all higher than 16.5 mmol L^−1^. After that, blood sugar levels were monitored continuously for 2 weeks. Before the establishment of the diabetic wound model, the mice fasted for more than 12 h. After isoflurane anesthesia, the back hair of each mouse was shaved and the back of each mouse was disinfected with 75% alcohol. Two round full‐thickness skin wounds (diameter = 8 mm) were made on the back of each mouse, and the back was sutured with silicone fixation rings to prevent the wound from shrinking. The mice were divided into four groups, one control group (untreated group) and three experimental groups. The control group was given only normal saline. The experimental group was divided into Alginate Dressing (positive control, commercially available hydrogel), CS/PL hydrogel, and CS/PL hydrogel + MOF, and the mice were treated with the above three hydrogels. It was worth noting that each of the above materials was only used once at the beginning. The above materials were injected into the wound area. At each prescribed treatment point (Day 3, 7, 14, and 21), wound photographs were taken so that the wound area could be calculated by Image J. At each time point, three mice in each group were killed, and the skin samples of the wound were taken for pathological analysis.

### Histological and Pathological Analysis

The wound tissue was dissected together with the adjacent healthy tissue, fixed with 4% paraformaldehyde, embedded in paraffin, and the section was 5 µm. The sections were stained with hematoxylin‐eosin (H&E) and the histological characteristics were observed. To further evaluate collagen deposition, sections were stained with Masson's Trichrome staining. Immunohistochemical staining was used to analyze inflammatory response and cell proliferation.

### Immunohistochemical Staining

The sections were successively dewaxed, rehydrated, heat‐induced antigen repair, and non‐specific binding was blocked. Primary antibody incubation (CD31:1/200; K6:1/200; CD206:1/200; Ki67:1/200; CCR7:1/200; Ly6G:1/600) and then use a PE‐labeled secondary antibody. After 5 min of DAB staining, the tissue sections were stained and observed under a microscope. Image J software was used to quantify the proportion of cells with positive staining in each visual field.

### Data Analysis

The findings were shown to be the means ± standard deviation (SD). All quantitative data were analyzed by one‐way ANOVA. Image J was used for image processing. ^*^ *P* < 0.05, ^**^ *P* < 0.01, or ^***^ *P* < 0.001 were considered statistically significant, ns = no significant.

## Conflict of Interest

The authors declare no conflict of interest.

## Supporting information



Supporting Information

## Data Availability

The data that support the findings of this study are available from the corresponding author upon reasonable request.
