# Supplementary material for: Thermosensitive Hydrogel Integrated with Bimetallic Nano‐Enzymes for Modulating the Microenvironment in Diabetic Wound Beds
Source: Adv Sci (Weinh). 2024 Dec 16;12(6):2411575. doi: 10.1002/advs.202411575 (PMC11809323; doi:10.1002/advs.202411575)
Supplement: Supplementary file 1 — Supporting Information [file ADVS-12-2411575-s001.docx]

**Thermosensitive Hydrogel Integrated with Bimetallic Nano-enzymes for Modulating the Microenvironment in Diabetic Wound Beds**

Chuwei Zhang ^1^, Xiaoyi Zhang ^1^,Fei Li ^1^, Bo Li, Mengnan Zhang, Wanqian Li,

Pan Zhuge, Jingye Yao, Yi Zhang, Shixuan Chen*, Yongjin Fang *, Chao Cai*

*C. Zhang, F. Li, B. Li, M. Zhang, W. Li, J. Yao, Y. Zhang, S. Chen, C. Cai*

Department of Burn and Plastic Surgery, Department of Wound Repair Surgery, Affiliated Hospital of Nantong University, Nantong, Jiangsu 226001, China

*X. Zhang*

Office of Clinical Drug Trial Institution, Affiliated Hospital of Nantong University

*C. Zhang, F. Li, B. Li, M. Zhang, W. Li, J. Yao, S. Chen, Y. Fang, C. Cai*

Zhejiang Engineering Research Center for Tissue Repair Materials, Wenzhou Institute, University of the Chinese Academy of Sciences, Wenzhou, Zhejiang 325000, China

*P. Zhuge, Y. Fang*

Department of Otolaryngology, Affiliated Jinhua Hospital, Zhejiang University School of Medicine, Jinhua, Zhejiang 321000, China

^1^ C. Zhang, X. Zhang, and F. Li contributed equally to this work.

*Corresponding Author:

[chensx@ucas.ac.cn](mailto:chensx@ucas.ac.cn) (S. Chen);

[5fyj@sina.com](mailto:5fyj@sina.com) (Y. Fang);

[caichao@ucas.ac.cn](mailto:caichao@ucas.ac.cn) (C. Cai)


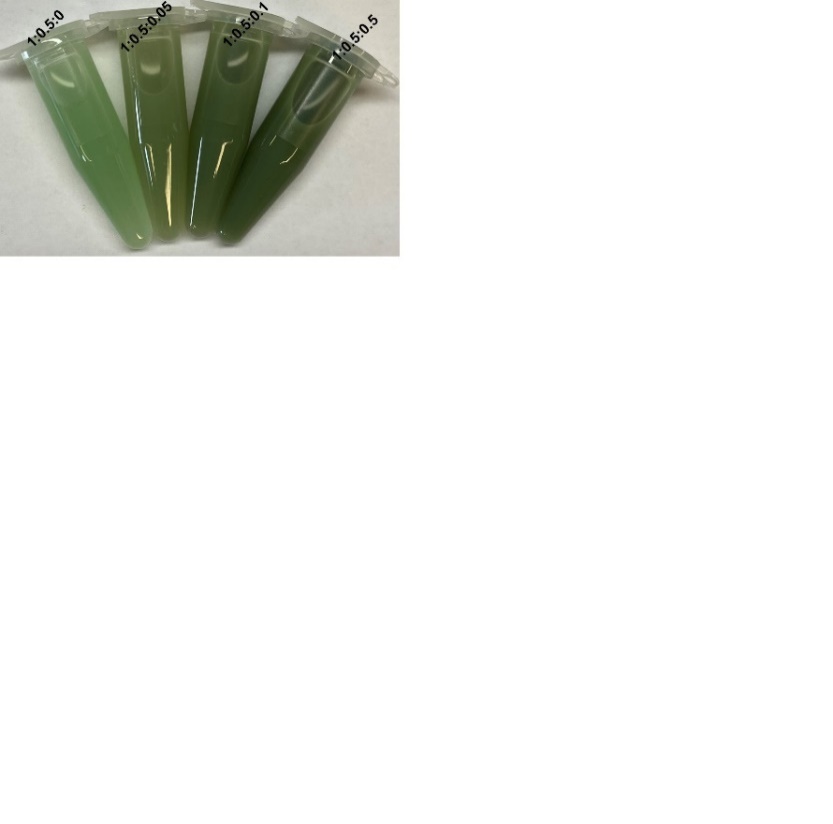


**Figure S1.** Images of the MOF solutions synthesized using varying ratios of raw materials, including different proportions of vitamin B6, copper acetate, and magnesium acetate.


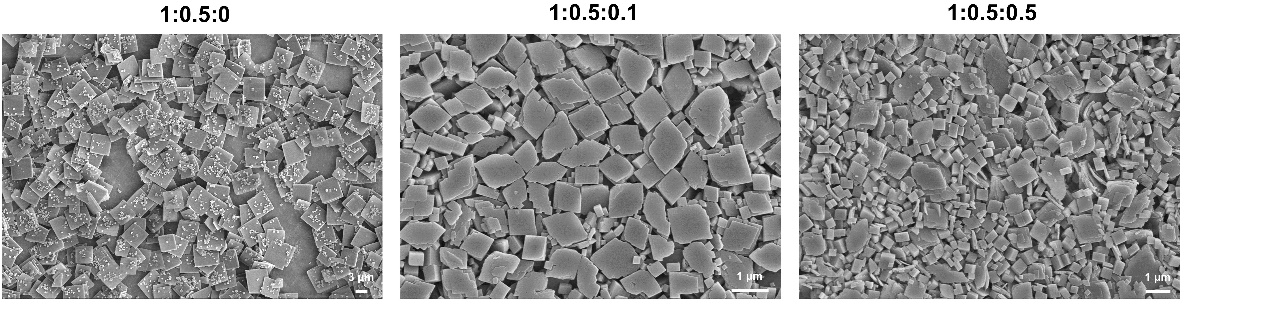


**Figure S2.** SEM images of the Cu/Mg-MOFs prepared with different proportions of vitamin B6, copper acetate, and magnesium acetate.

**Figure S3.** The content of different elements in the Cu/Mg-MOFs.


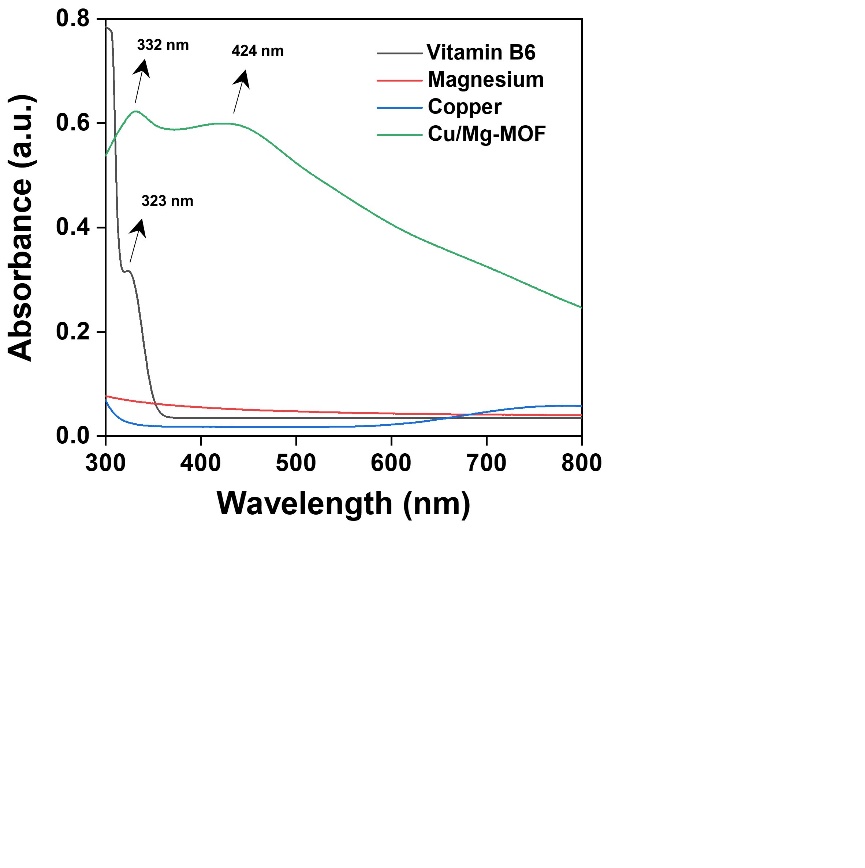


**Figure S4.** UV/vis absorption spectrum.


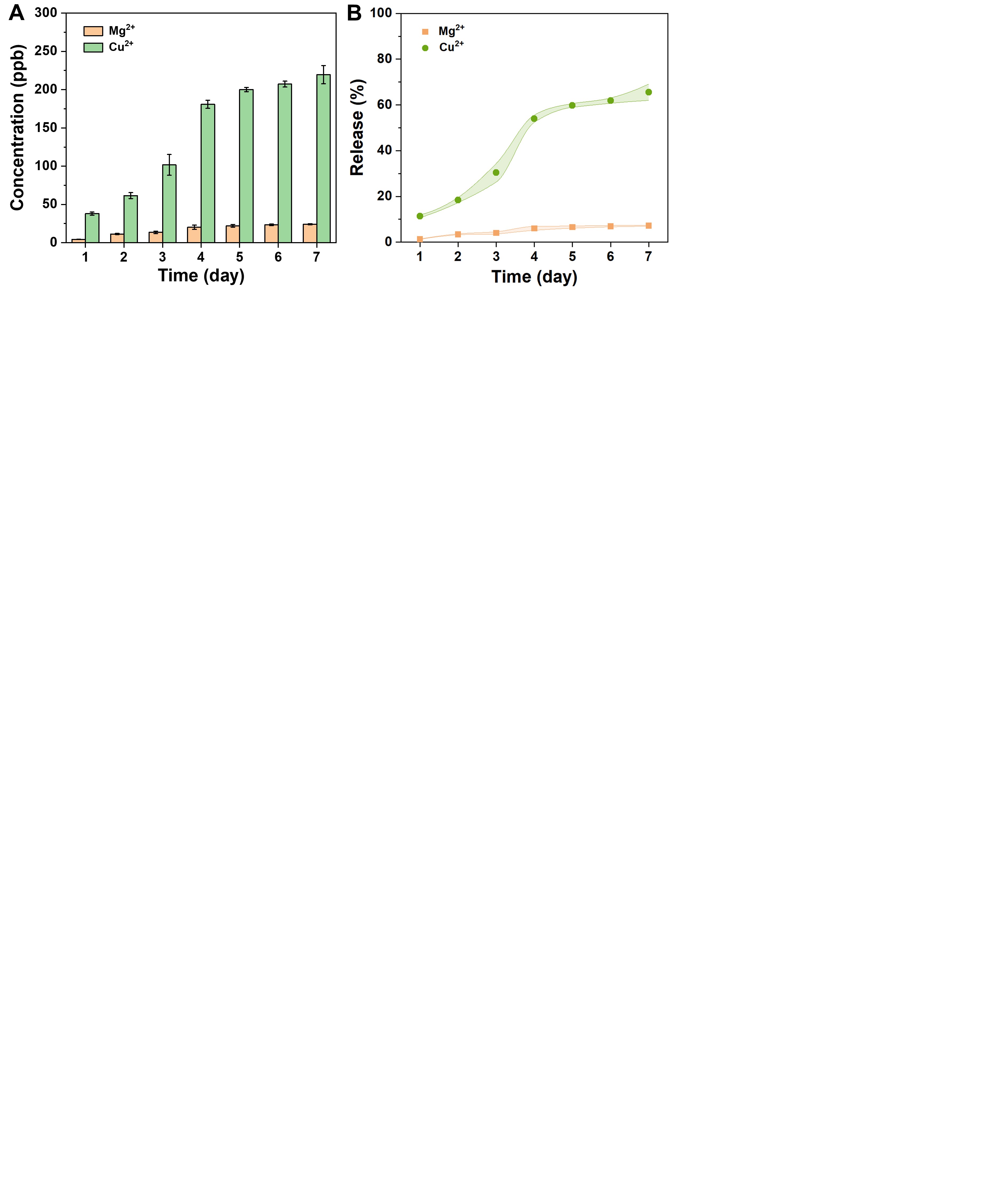


**Figure S5.** (A) The concentration of copper and magnesium ions released. (B) The release profile of copper and magnesium ions from the Cu/Mg-MOF@CS/PL hydrogel.


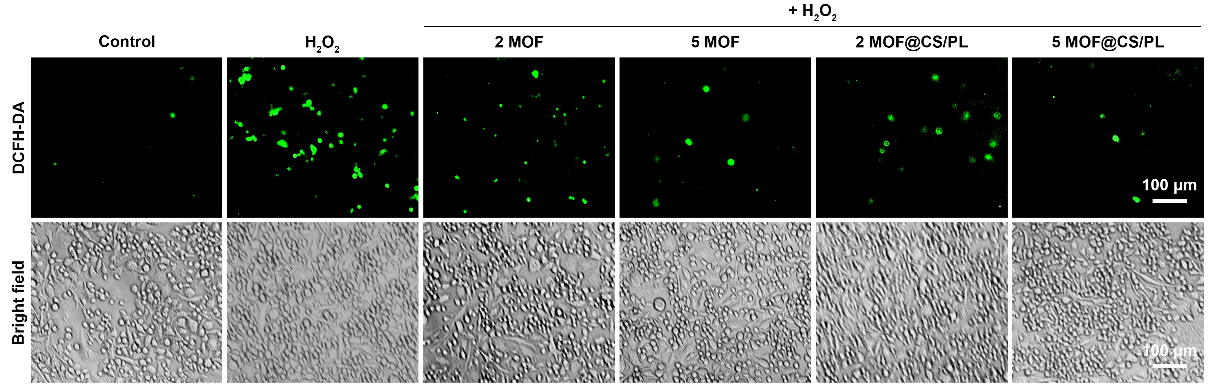


**Figure S6.** Representative ROS fluorescent staining images of RAW 264.7 with different treatments.


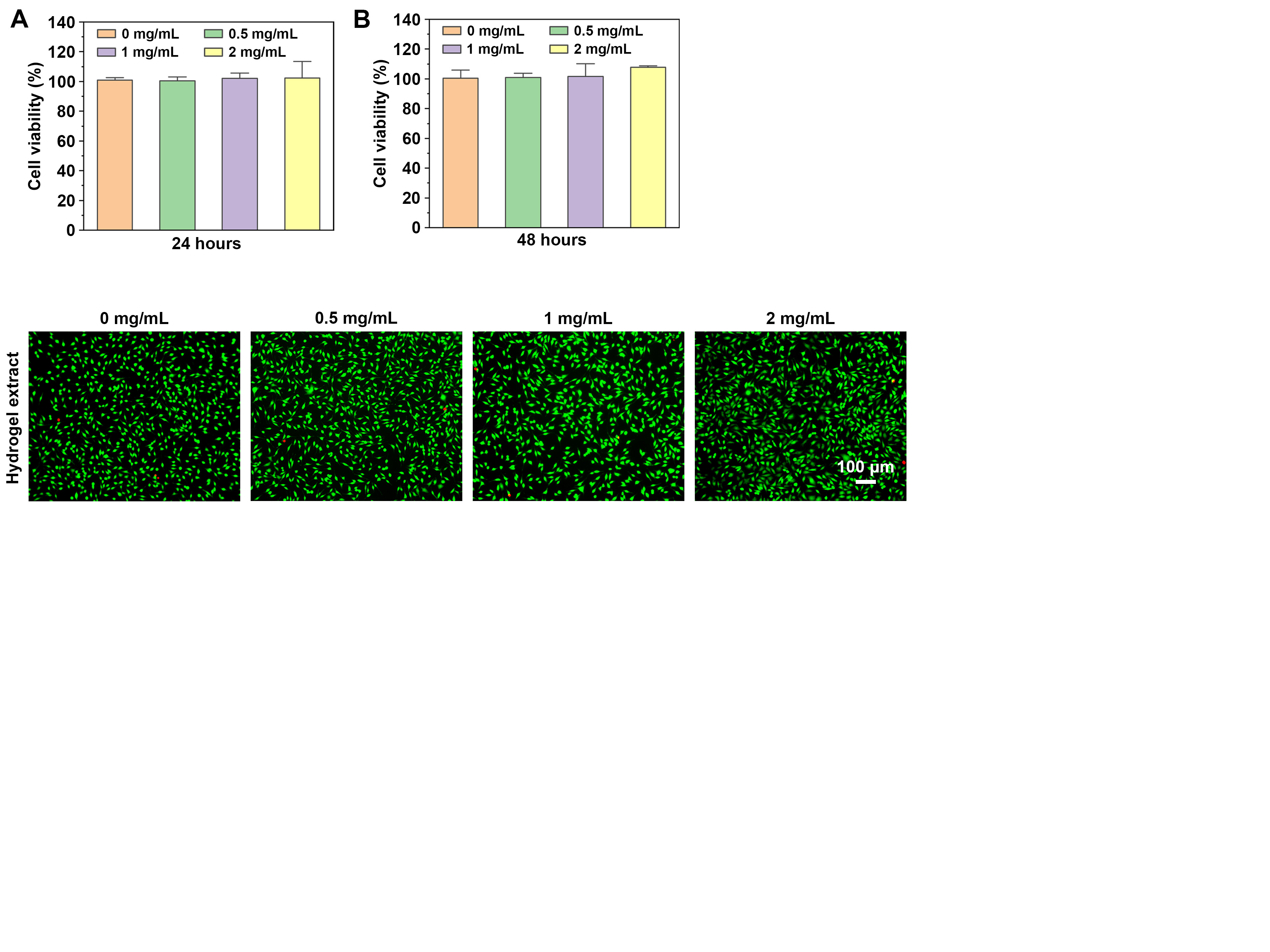


**Figure S7.** Cell viability of L929 cells after incubation with different concentrations of Cu/Mg-MOF@CS/PL hydrogel extracts at 24 hours (A) and 48 hours (B).


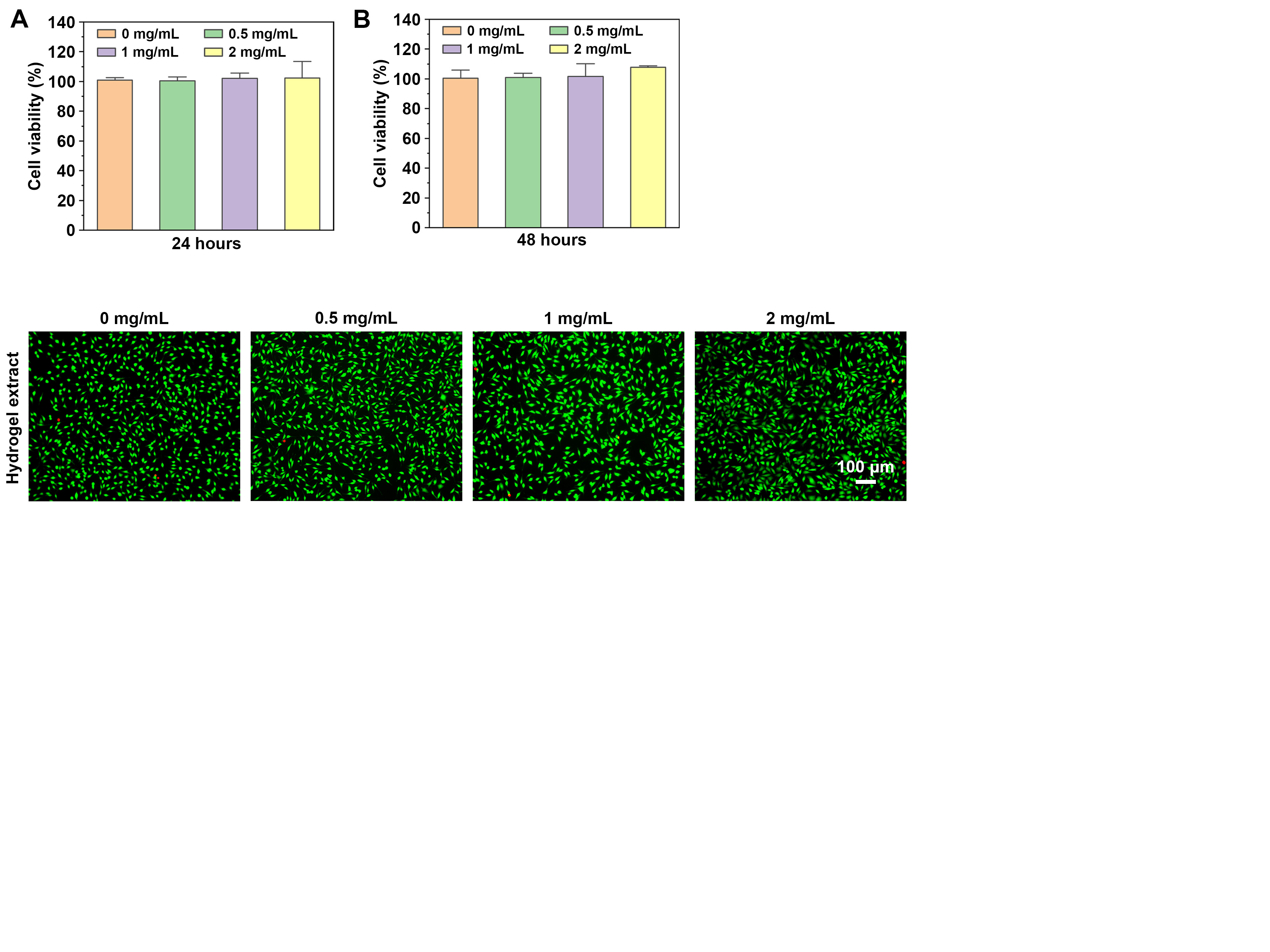


**Figure S8.** Live/dead staining of L929 cells after incubation with different concentrations of Cu/Mg-MOF@CS/PL hydrogel extracts.


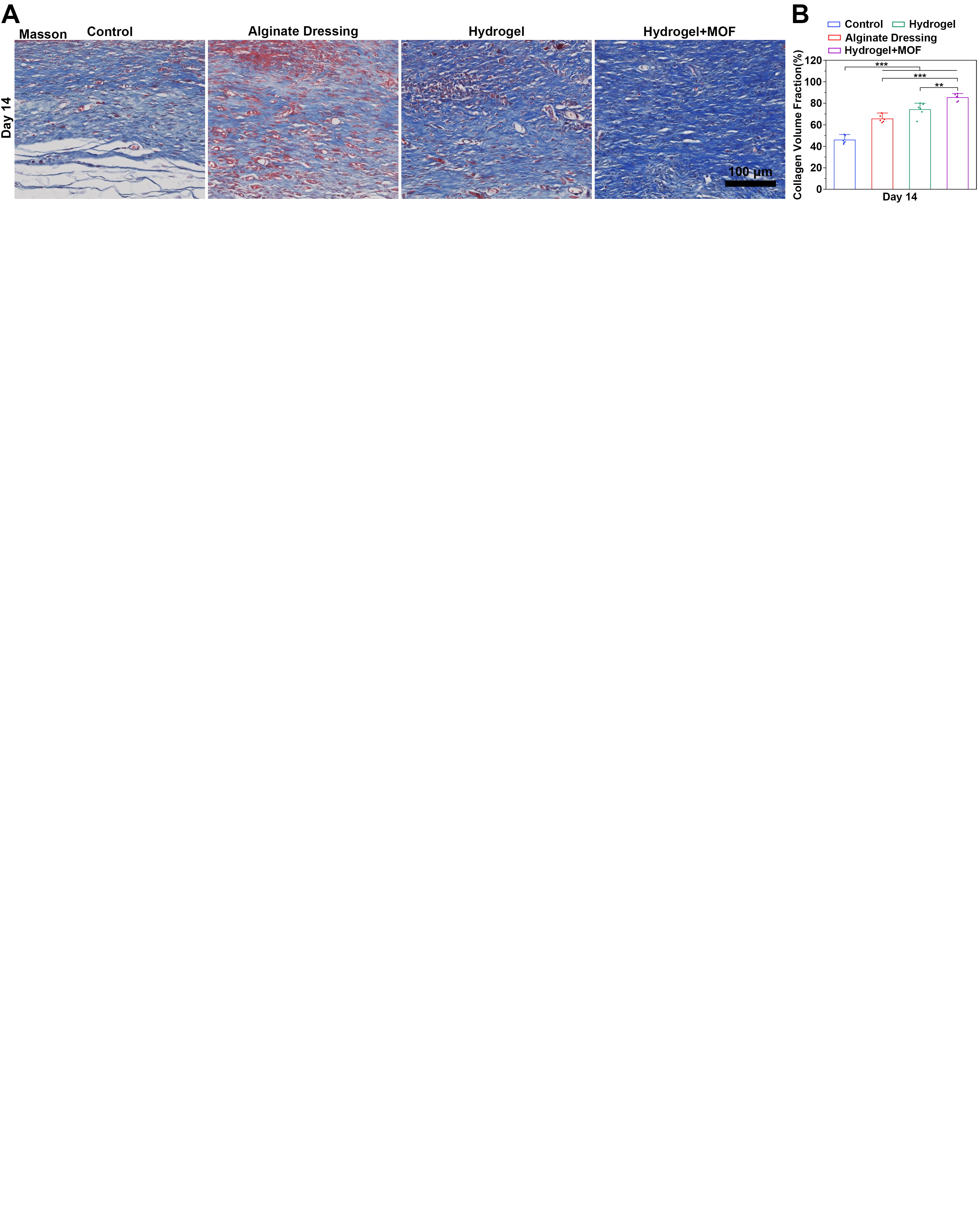


**Figure S9.** (A, B) Collagen deposition in the wound center area of control, alginate dressing, hydrogel, and hydrogel + MOFs groups after 14 days of treatment.
